# Plasmacytoid Dendritic Cells Capture and Cross-Present Viral Antigens from Influenza-Virus Exposed Cells

**DOI:** 10.1371/journal.pone.0007111

**Published:** 2009-09-22

**Authors:** Gabrielle Lui, Olivier Manches, Juliette Angel, Jean-Paul Molens, Laurence Chaperot, Joël Plumas

**Affiliations:** 1 Université Joseph Fourier, Grenoble, France; 2 Inserm, U823, Immunobiologie et Immunothérapie des Cancers, Grenoble, France; 3 EFS Rhône-Alpes, Laboratoire R&D, Grenoble, France; Federal University of São Paulo, Brazil

## Abstract

Among the different subsets of dendritic cells (DC), plasmacytoid dendritic cells (PDC) play a unique role in secreting large amounts of type I interferons upon viral stimulation, but their efficiency as antigen-presenting cells has not been completely characterized. We show here, by flow cytometry, with human primary blood PDC and with a PDC cell line, that PDC display poor endocytic capacity for soluble or cellular antigens when compared to monocyte-derived myeloid DC. However, immature PDC efficiently take up cellular material from live influenza-exposed cells, subsequently mature and cross-present viral antigens very efficiently to specific CD8+ T cells. Therefore, during viral infection PDC not only secrete immunomodulatory cytokines, but also recognize infected cells and function as antigen cross-presenting cells to trigger the anti-viral immune response.

## Introduction

Antigen presenting cells (APC) play a crucial role in the triggering and control of immune responses by their capacity to take up and process antigens, and to efficiently activate naive or memory T cells. Among APC, plasmacytoid dendritic cells (PDC) constitute a special subset of dendritic cells (DC) [1], [2], which were originally described as the main producers of type I interferons in viral infections [3], [4] and initiate immune responses.

Human PDC express the intracellular pattern-recognition receptors TLR7 and TLR9, allowing them to recognize viral or bacterial genetic material. Indeed, single-stranded RNAs were recently identified as natural ligands for TLR7/TLR8 [5], [6], whereas TLR9 recognizes unmethylated CpG oligonucleotides in endosomes or lysosomes after their internalization[7], [8]. Upon TLR ligation by natural or artificial TLR agonists, activated PDC secrete high amounts of IFNα, participating in the initiation of immune activation. Moreover, they rapidly become cytotoxic by acquiring TRAIL expression, so they can kill target cells expressing DR4 or DR5[9], receptors which are up-regulated upon viral infections[10]. Activated PDC are also involved in the selective recruitment of effector cells for both innate and adaptive immune responses[11].

PDC are able to differentiate into mature dendritic cells, when cultured with IL-3 and CD40-L [12], or upon viral stimulation [13]. Based on the initial stimulus, PDC stimulate allogeneic naïve CD4+ T lymphocytes and induce their differentiation in either Th2 [14], Th1 [15], IFNγ/IL10 producing T cells [16], or IL-10 producing T regulatory cells[17]. However it remains unclear whether they can prime a naïve T cell response [18], [19], although they can clearly restimulate anti-viral effector/memory CD4+ and CD8+ T lymphocytes by direct presentation [20], [21].

Besides direct priming, cross-priming is the mechanism of stimulation of CD8+ T cells by APC presenting exogenously-derived antigens; this is particularly important when viruses do not infect APC. Cross-presentation involves different steps; i) endocytosis of antigens, ii) their degradation and processing of the generated peptides onto MHC class I molecules, and iii) the expression of costimulatory molecules on APC surface. It has been suggested that human PDC, compared to myeloid DC, have a poor capacity to capture soluble or particulate antigen [12], [22]. PDC may enhance myeloid DC-mediated cross-presentation by means of IFNα secretion by PDC, which might favor this mechanism[23], but PDC's own ability to cross-present to CD8+ T cells is still a controversial and unresolved matter[24]. Indeed, the scarcity of PDC in peripheral blood mononuclear cells (0,5%)[25] and the difficulty to generate them in large numbers in vitro [26] render their study difficult. We have recently described a leukemic counterpart of PDC (LPDC for leukemic PDC) [27], which displays similar phenotypic and functional features to primary PDC [28]. A cell line has been generated from fresh LPDC, as described previously [9], and by using this cell line as a model, as well as primary PDC, we investigated endocytosis of soluble and cellular antigens by PDC. Our study aimed also at characterizing the ability of PDC to cross-present antigens taken up from whole cells during virus infection. Our data further demonstrate the prominent role PDC play in viral infections.

## Materials and Methods

### Reagents and phenotypes

The medium used for cell culture was RPMI 1640 Glutamax (GibcoBRL, Cergy-Pontoise, France) supplemented with 1 mM sodium pyruvate, 20 µg/ml Gentamycin, non-essential amino acids (referred to as complete medium), and 10% heat inactivated Fetal Calf Serum (FCS, Gibco).

Immunophenotyping was performed by flow cytometry on a FACScan (Becton Dickinson, Mountain View, CA) using direct or indirect labelling. The following FITC-, PE- or biotin- conjugated antibodies, as well as PC5-conjugated streptavidin, were purchased from Immunotech (Marseille, France): CD1a (clone BL6), CD4 (13B8.2), CD11b (Bear1), CD11c (BU15), CD18 (7B4), CD19 (J4.119), CD20 (B9E9), CD32 (2 E 1), CD36 (FA6-152), CD40 (mAb89), CD64 (22), CD80 (MAB104), CD83 (HB15A), HLA-ABC (B9.12.1), DR (B8.12.2), CD16 (3G8), CD86 (HA5.2B7), MR (3.29.B1.10). Antibodies against CD1c (M241) were purchased from Ancell, BDCA-2 (AC144), and BDCA-4 (AD5-17F6) from Miltenyi Biotech (Bergisch Gladbach, Germany), αvβ3 (LM609) and αvβ5 (P1F6) from Chemicon International (Temecula CA), CD1d (CD1d42) from Becton Dickinson, DC-SIGN (120507) from (R&D system, Lille, France), and CD123 (9F5) from Pharmigen (San Diego CA). The anti-mouse FITC-conjugated antibodies CD91 (A2MRα-2) was purchased from Dako (Glosgrup, Denmark).

Cell morphology was analyzed by microscopy on cytospins of cell suspension after staining with May-Grünwald Giemsa (Kit Ral 555). IFNα was measured by ELISA (PBL biomedical laboratories, Piscataway, NJ)

### Cells

The cell line GEN3 was generated from leukemic PDC [27], as described for the GEN2.2 cell line [9]. Tumor cells from the blood of the patient GEN were seeded on irradiated adherent mouse stromal cells (MS-5) in 10% FCS complete RPMI medium, without any cytokine. This cell line has been growing for months in our laboratory.

B lymphocytes were purified from healthy donors blood (B1 and B2) or from spleen biopsies obtained from a patient with idiopathic thrombocytopenic purpura (B3), by standard negative immunomagnetic selection with dynabeads (Dynal, Oslo, Norway). Purified B lymphocyte suspension contained more than 95% B cells, and were cryopreserved in liquid nitrogen and thawed immediately before use. B cells were chosen as a model as they represent a suitable target for influenza virus in vitro [29].

Myeloid dendritic cells (MoDC) were generated from monocytes of HLA-A2*0201 healthy donors as described [30]. Monocytes were purified from fresh blood by Rosette Sep isolation kit (Stem Cell Technologies, Meylan, France) and cultured for six days in complete medium supplemented with 10% of FCS, 500 U/ml GM-CSF (Leucomax, Schering-Plough, France) and 10 ng/ml IL-4 (Tebu Bio, Le Perray-en-Yvelines, France). At the end of the culture, MoDC were 100% CD1a+, CD14- and CD83-.

We isolated primary PDC from blood of healthy donors by using the positive selection BDCA-4 or BDCA-2 separator kit (Miltenyi Biotech), following the instructions of the manufacturer. The purity of enriched cell suspensions, was at least >90%, as assessed by FITC-conjugated BDCA-2 or BDCA-4, and PE-conjugated CD123. The immunophenotyping of PDC was done either on purified PDC, or in PBMC after gating on the BDCA-2 positive population.

### Endocytosis experiments

GEN3, primary PDC, or MoDC (0.5×10^6^ cells/ml) were incubated with 1 mg/ml Lucifer Yellow (LY, for liquid phase uptake) (Sigma Aldrich, Saint Quentin Fallavier, France), 0.1 mg/ml FITC-conjugated Dextran (Dex, for carbohydrate endocytosis) (MW 40,000-Molecular probes), and 0.1 mg/ml FITC-conjugated Ovalbumin (OVA, for protein endocytosis) (Molecular probes) for 2 hours at 37°C or at 4°C. After two washes in ice-cold 2% FCS HBSS (Hank's Balanced Salt Solution, GibcoBRL), cells were analyzed by flow cytometry. Mean fluorescence intensity (MFI) values were calculated by subtracting MFI of cells incubated with the tracers at 4°C, from the MFI at 37°C.

### PKH 26 staining and phagocytosis assays

For phagocytosis assays, B cells (40×10^6^ cells/ml) were stained with the lipophilic dye PKH26 (2 µM, (Sigma) (λ emission  = 576 nm)) following the indications of the manufacturer. After staining, B cells were then prepared as described in a previous work [31], in order to obtain apoptotic (γ-irradiated cells, ^137^ Cs source, 45 Gy), necrotic (heated for 45 min at 56°C), and Rituximab-opsonized cells (1 µg/ml).

For treatment with virus, B cells were incubated overnight with formaldehyde-inactivated influenza virus (strain A, New Caledonia/20/99 IVR116 (H1N1), Aventis Pasteur, Val de Rueil, France)(corresponding to 137 ng/ml hemagglutinin) in 10% FCS complete RPMI supplemented with 10 ng/ml IL-4 (Tebu Bio), and washed twice before use. These cells are further referred to as “flu-B cells”. A non-replicating virus was used throughout this study, in order to avoid the production of new virus that could interfere with cross-presentation experiments by directly infecting the antigen presenting cells. This virus is able to enter into target cells, since the nucleoprotein of the virus can be detected into the cytoplasm of B cells after incubation with the virus [29]. The non-infectious status of the virus was verified on eggs (data not shown).

In inhibition experiments, EDTA (2 mM), or cytochalasin D (10 µM) were added. For GEN3 maturation, CpG 2336 (12,5 µg/ml) (Coley Pharma, Ottawa Canada) was added for 24 h before addition of B cells.

For endocytosis assessment, APC (0.5×10^6^ cells/ml) were co-incubated for 2 hours at 37°C or 4°C with PKH26-stained B cells (0.75×10^6^ cells/ml). Endocytosis was stopped and conjugates were dissociated by adding EDTA (2 mM) on ice. APC were then stained: GEN3 with FITC-conjugated CD4 and CD36, MoDC with FITC-conjugated CD11b, and primary PDC with FITC-conjugated BDCA-2 and CD36. Simultaneously, B cells were stained with PECy-5-conjugated CD19 and CD20. After washing, PKH26 fluorescence was analyzed by flow cytometry gating on FITC-labeled APC. CD19 and CD20 positive APC were excluded from the analysis as they had likely formed conjugates with B cells but had not necessarily internalized cellular material.

### Evaluation of PDC maturation and IFNα secretion after endocytosis

GEN3 or purified BDCA4+ PDC were incubated or not with influenza virus for 24 hours, and cells were stained with FITC- or PE-conjugated CD40, CD80, CCR7 or HLA I mAb. To check the maturation and secretion of IFNα by PDC after contact with flu-B cells, B cells were exposed or not to virus for 18 hours, washed, and then incubated with GEN3 or purified BDCA4+ PDC. After a 24-hour co-culture, supernatant were collected, and their IFNα content was assessed by ELISA. Cells were stained with CD40, CD80, CCR7 mAb, and maturation was accessed by flow cytometry gating on GEN3 cells (according to their FSC/SSC profile) or on PDC (labeled with BDCA2 plus BDCA4 biotinylated mAb, and PeCy5-streptavidin).

### Cross-presentation assay

We assessed cross-presentation by GEN3, and primary PDC by measuring IFNγ released by influenza-specific T cells. Influenza-specific T cells were generated in one week-culture of purified autologous CD8+ T lymphocytes activated by influenza-treated and irradiated (30 Gy) HLA-A2+ MoDC [32], [33]. These influenza-specific cell lines contained 15 to 30% tetramer HLA-A2/Flu_58-66_ positive specific T cells; their specificity was checked using irrelevant and Flu-peptide pulsed T2 cells.

In the cross-presentation assay, allogeneic HLA-A2^neg^ B cells were treated with the virus during 18 hours and then extensively washed. In round-bottomed plates, in quadruplicates, 10^4^ GEN3 or primary PDC (HLA-A2^pos^) were co-incubated with 5×10^3^ irradiated (30Gy) B cells, treated or not with influenza virus, for 4 hours to allow endocytosis. 5×10^5^ influenza-specific T cells were then added, and after 48 hours, culture supernatants were recovered and their IFNγ content was measured using Cytokine Bead Array kit (Becton Dickinson, Le Pont de Claix, France) following the manufacturer's instructions.

## Results

### GEN3 is a PDC cell line derived from leukemic PDC

**Figure 1:**
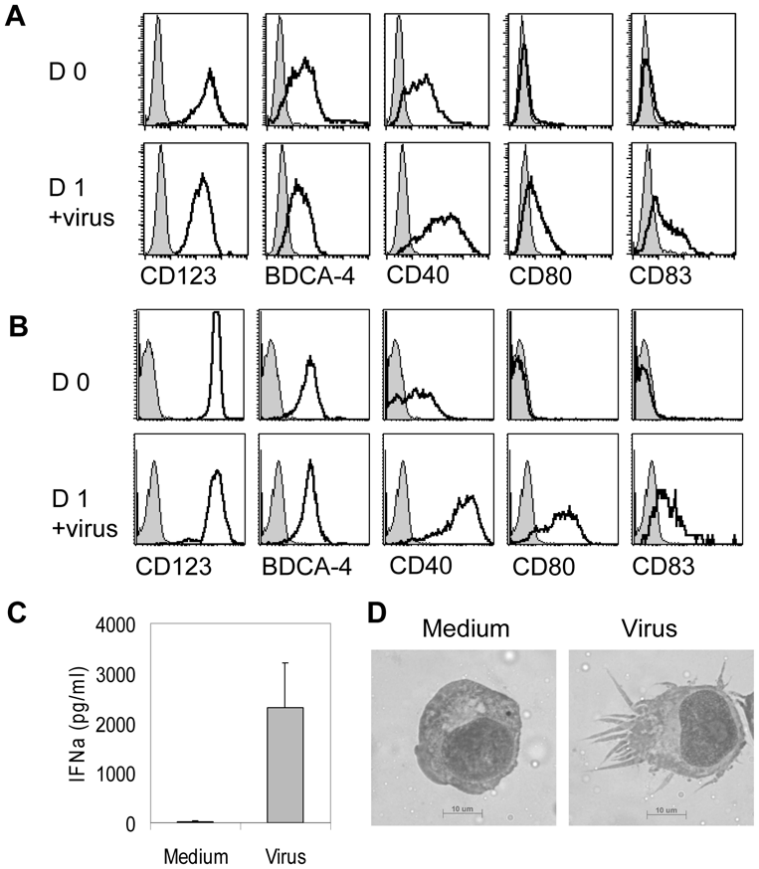
Characterization of the GEN3 plasmacytoid dendritic cell line. The LPDC-derived cell line GEN3 or primary purified PDC were incubated for 18 hours in the presence of formaldehyde-inactivated influenza virus. GEN3 cells (A) or primary PDC (B) were stained with indicated mAb (open curves) or isotype control (filled curves) at day 0 (upper panels) and day 1 (lower panels). At day 1 production of IFNα by GEN3 was measured by ELISA (C) (mean±SD of four experiments), and cell morphology was examined after May-Gründwald Giemsa staining of cell cytospins (D). Data are representative of 3 independent experiments.

GEN3 cell line was generated from leukemic plasmacytoid dendritic cells [27], [34]according to the method previously described[9]. They highly expressed CD123, BDCA-4, and CD40, whereas were negative for CD80 and CD83 (Figure 1A). Incubation with formaldehyde-inactivated influenza virus for 24 hours led to their maturation: GEN3 cells up-regulated CD40, CD80, and CD83 (Figure 1A), similarly to purified primary PDC (Figure 1B). GEN3 also secreted IFN-α (Figure 1C), and acquired a dendritic cell morphology (Figure 1D).

### PDC express few endocytic receptors

**Figure 2:**
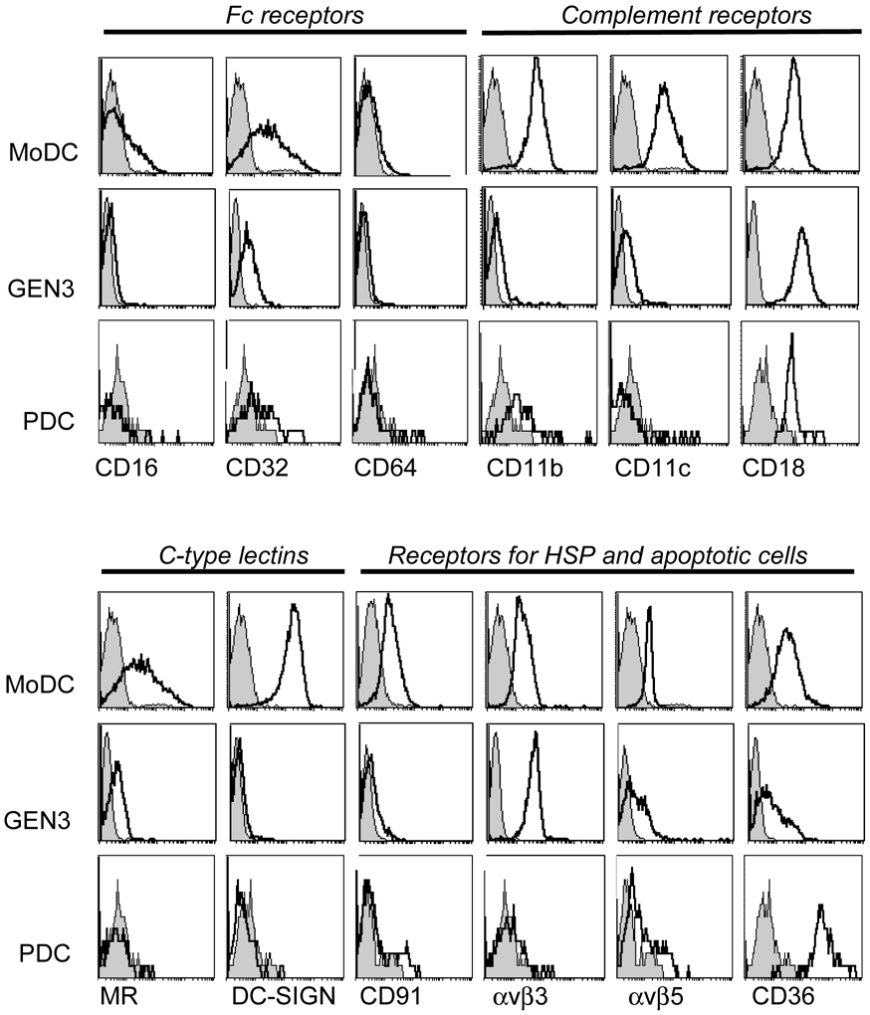
Assessment of the expression of receptors involved in antigen capture. Monocyte-derived dendritic cells (MoDC), GEN3 cells, and primary blood BDCA2+ PDC were stained with indicated mAb (open curves) or isotype control (filled curves). Data are representative of two independent experiments.

We then evaluated the surface expression of molecules involved in antigen capture (Figure 2). Primary PDC and GEN3 did not express the Fc receptors CD16 nor CD64, whereas a low expression of CD32 was detected. A weak expression of the C-type lectin mannose receptor (MR) was detected on GEN3, but not on primary PDC, whereas both primary PDC and GEN3 were negative for DC-SIGN and Langerin (data not shown). The β2 integrin CD18, a subunit of the complement receptors CR3 (CD11b/CD18) and CR4 (CD11c/CD18) was highly expressed by both primary PDC and GEN3, but CD11c and CD11b were hardly detectable. CD91 and calreticulin are involved in the uptake of apoptotic cells that are opsonized either with complement, collectins (SP-A and SP-D) or with heat shock proteins (review [35]). A low expression of CD91 was found on a small subset of primary PDC but not on GEN3. αvβ3 and αvβ5 are two integrins implicated in the clearance of apoptotic cells by DC and macrophages [32], [36], in association with CD36. Both GEN3 and primary PDC displayed a moderate expression of αvβ5 and a sharp expression of CD36. αvβ3 was present only on GEN3 cells. As a matter of comparison, the same phenotype was also performed on monocyte-derived dendritic cells, and we found that except for CD64, all the molecules studied were expressed on MoDC, and such a wide range of endocytic receptors may favor their ability to capture antigens. Conversely, PDC and GEN displayed a very restricted set of endocytic receptors.

### Endocytic capacity of PDC is weak

**Figure 3:**
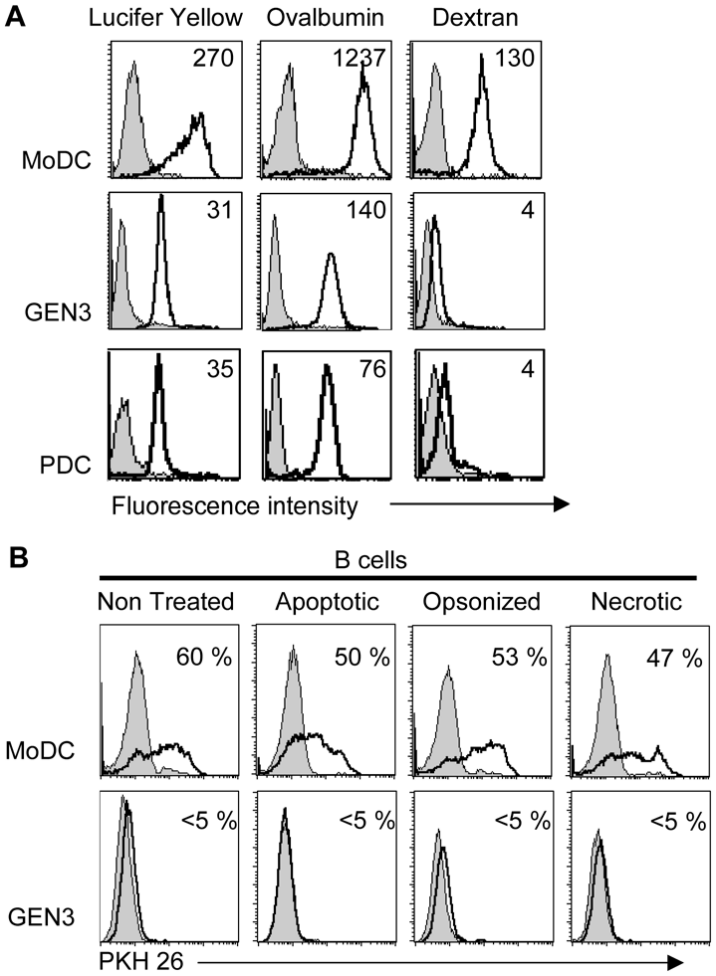
Measurement of soluble or cellular antigens uptake by flow cytometry. (A) Endocytosis of soluble tracers. MoDC, purified BDCA-4+ primary PDC or GEN3 cells were incubated for 2 hours at 4°C (filled curves) or 37°C (open curves) with Ovalbumin, Lucifer yellow and Dextran. Mean fluorescence intensity (MFI) values are indicated. Results are representative of two experiments. (B) Uptake of cellular material. PKH26-stained B cells were induced into apoptosis, necrosis, or were opsonized with CD20 mAb Rituximab or left untreated. They were then incubated with MoDC or GEN3 for 2 hours and stained as described in materials and methods section. PKH26 fluorescence, measuring cellular material uptake, was analyzed gating on MoDC (CD11b^pos^) or GEN3 (CD4^pos^CD36^pos^) not involved in cell conjugates (CD19^neg^CD20^neg^). Percentages of positive cells at 37°C (open curves), relative to incubation at 4°C (filled curves) are indicated. These data are representative of two independent experiments.

We assessed the ability of GEN3, primary PDC, and MoDC, to endocytose soluble antigens by using Lucifer Yellow (LY), FITC-conjugated Ovalbumin (OVA), and Dextran (Dex) (Figure 3A). Both GEN3 and primary PDC engulfed LY and OVA with the same efficiency, but less efficiently than MoDC (about ten-fold higher tracer capture was observed with MoDC compared to PDC). By contrast, primary PDC and GEN3 cells did not take up Dextran, whereas MoDC efficiently captured this potential ligand for mannose receptor.

We then evaluated the ability of PDC to take up cellular material from apoptotic, necrotic, opsonized and non-treated cells, in comparison to MoDC. To this end, we used a method that allows the detection of endocytosis with a high sensitivity and distinguishes between endocytosed or conjugated cells [29]. In these experiments, endocytosis was measured by flow cytometry using PKH26-labeled B-cells as target cells and appropriate FITC-conjugated mAbs to identify APC (Figure 3B). Conjugates were excluded using an additional labeling with PC5-conjugated CD19 and CD20. After 2 hours, at least half of the MoDC acquired cellular material from B cells, whatever the B cell treatment. By contrast, GEN3 remained PKH26^neg^, whatever the condition, suggesting their inability to capture opsonized or dead cells, or to perform “nibbling” of cells.

### PDC capture cellular material from influenza-treated cells

**Figure 4:**
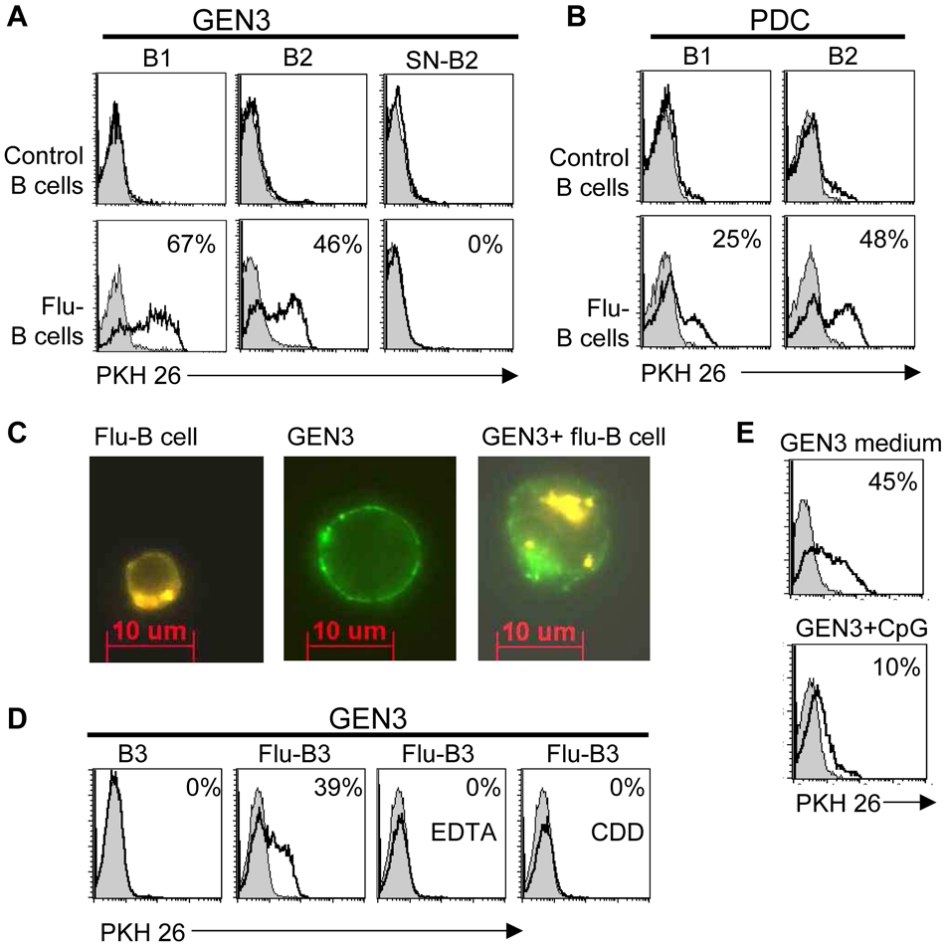
PDC internalized cellular material from influenza-treated B cells. (A and B) measurement of influenza-treated B cells uptake by flow cytometry. B cells (B1 and B2) were stained with PKH26, exposed (lower panels) or not (upper panels) to virus for 18 hours, extensively washed and then incubated with purified PDC or GEN3 for 2 hours at 4°C (filled curves) or 37°C (open curves). PKH26 fluorescence was evaluated as in Figure 3, gating on primary PDC and GEN3 not involved in conjugates with B cells. As control, GEN3 were also incubated following the same procedure for two hours with the supernatant of 18-hour flu-B cell culture (SN-B2). (C) Fluorescence microscopy imaging of B cell material by GEN3. PKH26+ flu-B cells appear yellow and GEN3 were stained in green with FITC-conjugated CD4 and CD36. (D) Membrane capture is Ca^2+^and actin-dependent. Cellular uptake was measured as described in Fig. 3A, at 4°C (open curves) or 37°C (filled curves) in the presence or absence of EDTA or cytochalasin D (CDD). (E) Maturation down-regulated cell capture by PDC. GEN3 were matured in presence of CpG for 24 h (lower panel) or not (upper panel) and incubated with B cells exposed (open curves) or not (filled curves) to virus for 18 hours.

Because PDC are involved in anti-viral immune responses, we sought to determine whether GEN3 cells capture cellular antigens from virus-infected cells. We used B cells incubated with formaldehyde-inactivated non-replicating influenza virus as a model of virus-containing cells, henceforth referred to as “flu-B cells”. As shown in Figure 4A, cellular material from flu-B cells was efficiently taken up by GEN3 (46 and 67% of GEN3 were PKH26^pos^ in the two representative experiments shown). As expected, non-treated B cells were not captured. Interestingly, primary purified BDCA4+ PDC were also able to capture cellular material from flu-B cells (25 and 48% of PDC were PKH26^pos^ in the two representative experiments shown) (Figure 4B). So, PDC seems to specifically take up cell-derived material from virus-containing surrounding cells. Microscopic observation of cell fluorescence showed that GEN3 took up small membrane-containing fragments from virus-treated cells (Figure 4C). This uptake of cellular material from flu-B cells required actin polymerization and divalent cations, as demonstrated by inhibition of uptake with cytochalasin D and EDTA, respectively (Figure 4D). This endocytosis was not due to the capture of PKH26^pos^ vesicles such as exosomes or other kinds of membrane shed particles. Indeed, when GEN3 were incubated for two hours with the supernatant of 18-hour flu-B cells culture, they remained PKH26^neg^ (Figure 4A).

To test whether cell maturation influenced this particular endocytic capacity of PDC, we assessed capture after activation of GEN3 cells with a TLR9 agonist. Figure 4E shows that in the presence of CpG A (2336), the capacity of GEN3 cells to take up cellular material from flu-B cells was inhibited, i.e. the percentage of PKH26^pos^ GEN3 decreased from 45% (for immature GEN3) to less than 10% (for mature GEN3).

### Endocytosis of flu-B cells activates PDC and induces cross-presentation of viral antigens

**Figure 5:**
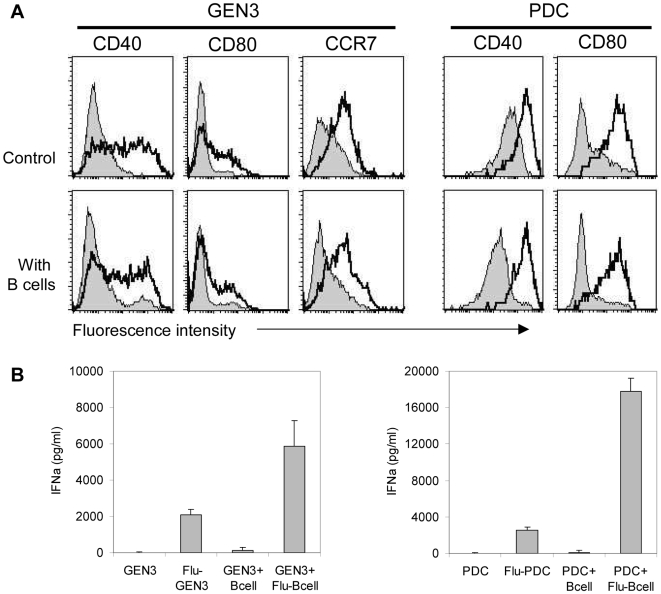
Activation of PDC upon exposure to flu-B cells. (A) In upper panels, GEN3 or purified PDC were incubated (open curves) or not (filled curves) with influenza virus for 24 hours, as positive control of their maturation, and cells were stained with indicated mAb. In lower panels, B cells were exposed (open curves) or not (filled curves) to virus for 18 hours, washed, and then incubated with GEN3 or purified PDC. After a 24-hour co-culture, cells were stained with indicated mAb, and maturation was assessed by flow cytometry after gating on GEN3 cells (according to their FSC/SSC profile) or on BDCA2^pos^ BDCA4^ pos^ PDC. Data are representative of two independent experiments. (B) GEN3 or purified PDC were incubated with or without influenza virus, B cells or Flu-B cells for 24 hours, and then IFNα levels in supernatants were measured by ELISA. Data represent mean±SD of 2 experiments performed in duplicates.

We next analyzed the consequences of flu-B cells capture on PDC maturation. When GEN3 were directly treated with the inactivated virus, they upregulated CD40, CD80, CCR7 (Figure 5A, upper panels), and HLA-I expression (data not shown). Interestingly, after endocytosis of flu-B cells, GEN3 were found activated to a similar extent (Figure 5A, lower panels). Primary purified BDCA4+ PDC also matured after endocytosis of flu-B-cells, upregulating CD40 and CD80 up to intensity comparable to that observed after direct contact with the virus. (Figure 5A, right panels). Therefore, PDC became activated following the capture of cellular material from virus-treated cells. Since the supernatant of flu-B cells did not induce the maturation of PDC (data not shown) the involvement of free viral particles was excluded. We also found significant IFNα secretion by GEN3 and primary PDC after endocytosis of flu-B cells (Figure 5B).

**Figure 6:**
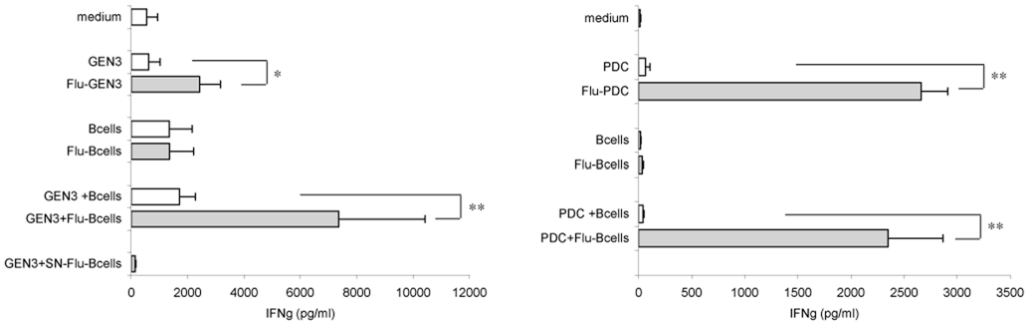
PDC cross-present viral antigens derived from flu-B cells to influenza-specific CD8+ T lymphocytes. HLA-A2^neg^ B cells exposed (filled bars) or not (open bars) to inactivated influenza virus for 18 hours were washed and incubated with HLA-A2^pos^ APC (either GEN3 (A) or PDC (B)), for four hours, followed by the addition of influenza-specific CD8+ T lymphocytes (recognizing flu_58-66_ influenza matrix peptide in the context of HLA-A2 molecules). After a 2-day co-culture, T cell activation was assessed by measuring the IFNγ content of the culture supernatants. In control wells, PDC were directly treated or not with the virus, or were omitted (medium). (A) Values are mean + SD of six independent experiments, except for the last control bar (SN-Flu-B cells), mean of two experiments, where Flu-B cells were washed extensively, and further incubated for 24 hours to recover the supernatant which was subsequently added to influenza-specific T cells. (B) Values are representative of two experiments and shown the mean + SD of quadruplicate wells. * P<0.05, ** P<0.005 by Student's t test.

We next analyzed the ability of PDC to activate influenza-specific T cells. The activation of HLA-A2^pos^ influenza-specific CD8+ T cells by both kinds of PDC was assessed by measuring IFNγ release by T cells. As expected, when virus-treated GEN3 (Figure 6A) or primary purified BDCA2+ PDC (Figure 6B) were used, IFNγ secretion was observed, confirming the ability of PDC to perform direct presentation of viral antigens. Cross-presentation of viral antigens by PDC was then examined by incubating GEN3 or primary PDC (both HLA-A2^pos^) with B cells or flu-B cells (HLA-A2^neg^), prior to addition of T cells. Strikingly, GEN3 (Figure 6A) as well as primary PDC (Figure 6B) pulsed with flu-B cells induced a highly significant increase of IFNγ secretion by T cells, when compared to non-treated B cells (p<0,005, student t test). This increase was not due to direct T cell activation by flu-B cells, as flu-B cells alone did not induce any IFNγ increase. Moreover, T-cell activation driven by GEN3 pulsed with the supernatant of 48h-virus-treated B cell culture was found negative, ruling out the involvement of free viral particles. These data demonstrate that PDC capture viral antigens from virus-exposed B cells and subsequently cross-present these antigens to specific CD8+ T cells. This highly significant result indicates a striking specific role of PDC in activation of T cell responses in the context of viral infection.

## Discussion

PDC are involved early in innate phases of viral immunity through the secretion of large amounts of IFNα[1], [2], and TRAIL-mediated cytotoxicity[9]. Previous studies have clearly established that, upon exposure to viruses, PDC mature and acquire costimulatory molecules (reviews[1], [2]), so that infected PDC become able to present viral antigens to CD8+ T cells[20], [37]. These data suggested the involvement of PDC in the initiation of adaptive immunity in the context of viruses that directly infect PDC. Here, we evaluated the capacity of PDC to perform cross-presentation, i.e. to acquire, process, and present exogenously-derived antigens on HLA class I molecules to activate specific CD8+ T cells[38]. The physiological essential function of cross-presentation in viral infections has been demonstrated in the context of immune responses to viruses that do not infect APC[39]. Recent studies in human reported contradictory data regarding the capacity of PDC to cross-present antigens[37], [40].

We took advantage here of a cell line derived from leukemic PDC (LPDC[27], [28]), GEN3, as a model to study the uptake of antigens by PDC, and their capacity for cross-presentation. According to previous studies [22], [41], PDC and GEN3 displayed a weak capacity to capture dextran, which correlates with the absence of mannose receptor and DC-SIGN expression, whereas they took up the liquid phase tracer Lucifer Yellow, and a soluble protein such as ovalbumin, although less efficiently when compared to MoDC. We have shown that GEN3 expressed quite the same pattern of endocytic receptors as primary PDC, the most striking difference being the expression of αvβ3 on GEN3 but not on primary PDC. On both cells, we found a weak expression of CD32 (as previously described [42]), and a faint expression of CD11b and CD11c. However, CD32 did not allow the capture of Rituximab-opsonized cells by GEN3 albeit it was shown to mediate the capture of immune complexes containing DNA in the context of human lupus [43], or KLH after vaccination in melanoma patients[44]. According to previous work[45], PDC did not take up cellular material from live, apoptotic, or necrotic B cells even though they expressed CD36 and low levels of αvβ5, two receptors involved in the capture of dying cells. This was in contrast with MoDC, which efficiently endocytosed B-cell derived material from all the different sources of B cells. This phenomenon may be due to the expression of more diverse endocytic receptors on MoDC allowing them to capture apoptotic or necrotic cells (CD91, CD36, αvβ3, αvβ5), opsonized cells (CD16 or CD32), or to perform the so-called “nibbling” of small vesicles from live cells [46]–[48]. Altogether, MoDC are undoubtedly more efficient than PDC at endocytosing cells.

Interestingly, our results show an efficient transfer of cellular material from influenza-treated B cells (flu-B cells) to PDC, in an actin and Ca^2+^ dependent manner. We documented this uptake by fluorescence microscopy, and found that most PDC contained fragments of virus-treated cells. The functional consequences of this endocytosis by PDC were then examined. Upon exposure to flu-B cells of GEN3 or primary PDC secreted IFNα and matured, as evidenced by acquisition of costimulatory molecules. The up-regulation of CCR7 suggests that after the encounter with infected cells in periphery, and capture of cell-derived antigenic material, PDC could traffic to T cell zones in the lymph nodes and present acquired antigens. Furthermore, our study provides further evidence that following uptake and maturation, PDC are able to cross-present viral antigens captured from virus-exposed cells to specific T cells. The first demonstration of the ability of PDC to cross-present viral antigens taken from infected cells was recently performed by Hoeffel at Al[49], in a model where PDC cross-presented to specific T cells antigens taken from apoptotic HIV-infected cells. This mechanism reveals a highly relevant role for PDC in eliciting anti-viral immune responses. Indeed, during a natural infection, DC are not necessarily the primary infected cells, and admittedly virally infected non-APC are unable to stimulate CD8+ T cell immunity. Moreover, many viruses such as HIV, measles virus or HCMV infect DC, and have evolved mechanisms to subvert DC function, by disturbing their maturation or ability to secrete cytokines[50]–[52]. Here, by their ability to engulf viral antigens from virus-containing cells without being themselves infected, PDC could acquire and present viral antigens, avoiding virus-induced subversion of their functions.

Our results show that after capture, viral-antigens are processed and presented into the MHC class I pathway. How do PDC detect the infection, and acquire cell-derived fragments? Among flu-B cells, some died in vitro, however, in our hands PDC engulfed neither necrotic nor apoptotic cells (our data and [45]). Therefore, recognition of influenza-treated living cells by PDC likely did not rely on dead cell capture, contrary to the model recently described in MoDC by Frleta et Al [53]. Flu-B cells expressed viral hemagglutinin on the B cell surface (data not shown), a molecule that could allow PDC recognition of virus-treated cells. A potential receptor is NKp44, which recognizes influenza hemagglutinin[54], but it is expressed on PDC only after activation[55]. Since mature GEN3 lost their endocytic capacity, the receptor involved is likely down regulated during PDC maturation. Further studies are required to determine whether the recognition of infected cells by PDC relies on virus- or endogenous cell-derived signals, and what kind of receptor on PDC is involved.

The efficiency with which viral antigens from flu-B cells were cross-presented may be due to the high efficiency of their uptake by PDC. It is also possible that IFNα secreted by PDC in association with other unknown factors may enhance the cross-presentation of internalized antigens[23], through a still undetermined mechanism. IFN-β, not measured in our study, could also be involved, as this pro-inflammatory cytokine secreted by various kinds of infected cells has been described to prime DC, enhancing their activation in response to influenza virus[56]. Alternatively, engagement of TLR (likely TLR7, expressed by PDC[57] and involved in influenza virus recognition[5]) on PDC may influence phagosome maturation in PDC [58], and favor the generation of viral peptides for cross-presentation. Whatever the mechanism, cross-presentation of viral antigens was at least as efficient as direct presentation by PDC exposed to influenza virus.

In conclusion, our study identifies a role for PDC as a central professional APC during viral infections, by being extremely sensitive not only to direct viral exposure, but also to infection of bystander cells. We suggest that PDC play a major role in the control of viral infection through IFNα secretion and TRAIL expression during the innate phase of immune response, and by the further activation CD8+ T cell adaptive immunity through cross-presentation of viral derived antigens captured from infected cells. Thus, the role of PDC as a major link between innate and adaptive immunity is further emphasized.
